# Anti‐stromal treatment together with chemotherapy targets multiple signalling pathways in pancreatic adenocarcinoma

**DOI:** 10.1002/path.4727

**Published:** 2016-05-25

**Authors:** Elisabete F Carapuça, Emilios Gemenetzidis, Christine Feig, Tashinga E Bapiro, Michael D Williams, Abigail S Wilson, Francesca R Delvecchio, Prabhu Arumugam, Richard P Grose, Nicholas R Lemoine, Frances M Richards, Hemant M Kocher

**Affiliations:** ^1^ Centre for Tumour Biology, Barts Cancer InstituteQueen Mary University of LondonLondonUK; ^2^ The University of Cambridge Cancer Research‐UK Cambridge InstituteLi Ka Shing Centre, Robinson WayCambridgeUK; ^3^ Centre for Molecular OncologyBarts Cancer Institute, Queen Mary University of LondonLondonUK; ^4^ Barts and The London HPB CentreThe Royal London HospitalBarts Health NHS TrustLondonUK

**Keywords:** gemcitabine, all‐trans‐retinoic acid, quiescence, pancreatic stellate cells, collagen, fibronectin

## Abstract

Stromal targeting for pancreatic ductal adenocarcinoma (PDAC) is rapidly becoming an attractive option, due to the lack of efficacy of standard chemotherapy and increased knowledge about PDAC stroma. We postulated that the addition of stromal therapy may enhance the anti‐tumour efficacy of chemotherapy. Gemcitabine and all‐trans retinoic acid (ATRA) were combined in a clinically applicable regimen, to target cancer cells and pancreatic stellate cells (PSCs) respectively, in 3D organotypic culture models and genetically engineered mice (LSL‐Kras^G12D^^/+^;LSL‐Trp53^R172H^^/+^;Pdx‐1‐Cre: KPC mice) representing the spectrum of PDAC. In two distinct sets of organotypic models as well as KPC mice, we demonstrate a reduction in cancer cell proliferation and invasion together with enhanced cancer cell apoptosis when ATRA is combined with gemcitabine, compared to vehicle or either agent alone. Simultaneously, PSC activity (as measured by deposition of extracellular matrix proteins such as collagen and fibronectin) and PSC invasive ability were both diminished in response to combination therapy. These effects were mediated through a range of signalling cascades (Wnt, hedgehog, retinoid, and FGF) in cancer as well as stellate cells, affecting epithelial cellular functions such as epithelial–mesenchymal transition, cellular polarity, and lumen formation. At the tissue level, this resulted in enhanced tumour necrosis, increased vascularity, and diminished hypoxia. Consequently, there was an overall reduction in tumour size. The enhanced effect of stromal co‐targeting (ATRA) alongside chemotherapy (gemcitabine) appears to be mediated by dampening multiple signalling cascades in the tumour–stroma cross‐talk, rather than ablating stroma or targeting a single pathway. © 2016 The Authors. *The Journal of Pathology* published by John Wiley & Sons Ltd on behalf of Pathological Society of Great Britain and Ireland.

## Introduction

Combination chemotherapy regimens consisting of oxaliplatin, irinotecan, fluorouracil, and leucovorin (FOLFIRINOX) 1 or nab‐paclitaxel with gemcitabine 2 have resulted in increased median overall survival in pancreatic cancer (PDAC), compared with gemcitabine alone, which is the currently approved and widely used palliative mono‐therapy 3. However, gains have been marginal, and this may well be because desmoplasia remains largely unaltered with therapy 4.

PDAC is characterized by a pronounced desmoplastic stroma resulting from the activation of quiescent pancreatic stellate cells (PSCs) 5. This stroma creates a hypoxic microenvironment that, paradoxically, promotes both tumour growth and metastatic spread while inducing vascular collapse, thus creating a barrier to the passage of therapeutic agents 6; altogether, these features make the cellular desmoplastic stroma an appealing therapeutic target. In a genetically engineered mouse model, *LSL‐Kras^G12D/+^;LSL‐Trp53^R172H/+^;Pdx‐1‐Cre* (KPC) mice, the pharmacological inhibition of the sonic hedgehog signalling pathway, in combination with gemcitabine, produced variable results dependent on disease stage 7, 8, but provided proof of concept that stromal targeting was feasible. However, stromal ablation leads to a biologically more aggressive form of PDAC 8, 9, indicating that attention to the spatio‐temporal aspects 4 of the tumour–stroma cross‐talk may be critical for its effective targeting 10.

PSCs play a central role in desmoplastic stroma 11, 12. Previously, we demonstrated that restoring the quiescent state of PSCs, by replenishing their physiological retinol depots using the pleiotropic agent all‐*trans* retinoic acid (ATRA), halted tumour progression through targeting multiple tumour–stromal signalling cascades 13, 14, a notion recently supported by targeting the vitamin D receptor 15. In the present report, we use combination therapy to target pancreatic cancer cells and their supporting stroma in *in vitro* and *in vivo* PDAC models to demonstrate the efficacy of this strategy.

## Materials and methods

### Organotypic cultures

Short tandem repeat (STR) profiled cancer (Capan‐1, AsPC1) and stellate (PS1) cells were cultured, and pancreatic organotypic cultures were constructed as described elsewhere 11, 16, 17, 18. The two cancer cell lines represent a spectrum of PDAC differentiation 11, 13, 18. The pancreatic stellate cell line used was PS1, which was derived from a normal pancreas (rejected for transplantation) donated by the UK human tissue bank [Ethics approval; Trent MREC (/MRE04/)]. The cells were isolated using the outgrowth method 19, 20, followed by immortalization by ectopic expression of human telomerase reverse transcriptase (hTERT) 21, and verified as being of stellate cell origin by positive immunostaining for desmin, vimentin, smooth muscle α‐actin (α‐SMA), and glial fibrillary acidic protein, and ability to store vitamin A 13.

In contrast to previous reports 13, 17, we allowed the cancer/stellate interaction to be established for 10 days 11, before commencing treatment, to mimic an established tumour analogue. The cancer/stellate cell ratio was 1/2, as determined previously, providing the most aggressive, invasive phenotype within this model which mimicked histological features of advanced human cancer 11. Multiple biological and technical replicates performed by two independent researchers ensured reproducibility. Two treatment cycles were given, as per the human clinical protocol 3, 22. Briefly, treatment of organotypic cultures was performed daily with ATRA (R2625; Sigma, St Louis, MO, USA) at 1 µm or weekly with gemcitabine (2',2'‐difluoro 2'‐deoxycytidine, dFdC) (Eli Lilly, Indianapolis, IN, USA), either at 100 nm (Capan‐1/PS1) or 400 nm (AsPC1/PS1), or with the gemcitabine/ATRA combination, or with respective vehicles. Organotypic cultures were harvested on day 24, fixed in 10% neutral buffered formalin (BAF‐0010‐03A; CellPath Ltd, Newton, Powys, UK), embedded in paraffin, and cut into 4‐µm sections. Each experiment had three technical replicates and at least three biological repeats.

### Treatment of KPC mice

All animal work was done in accordance with the UK Animals (Scientific Procedures) Act 1986, revised by the Amendment Regulations 2012 (SI 2012/3039) to transpose European Directive 2010/63/EU, with approval from the local Animal Welfare and Ethical Review Body, and following the 2010 guidelines from the UK Coordinating Committee on Cancer Research 23. Compound mutant KPC mice with mature, established tumours were enrolled at a median age of 180 days and used as previously described 7, 13. ATRA was dissolved to 25 mg/ml in dimethyl sulphoxide, further diluted to 2.98 mg/ml in (2‐hydroxypropyl)‐β‐cyclodextrin (H5784; Sigma‐Aldrich), and finally to 1.5 mg/ml with sterile‐filtered tap water. This ATRA solution was administered orally to mice at 15 mg/kg daily for 7 days 13. Gemcitabine was injected intraperitoneally at 100 mg/kg on days 0, 3, and 7 7. Tumour volumes were measured by ultrasound 2 days before beginning treatment, and mice bearing tumour volumes of ∼250 mm^3^ (supplementary material, Table S1) were selected for the study. Tumours were harvested 7 days after beginning treatment and immediately submerged in formalin for 24 h, followed by embedding in paraffin blocks for further sectioning and immunostaining analysis. The primary endpoint of the study was drug efficacy as measured by a number of surrogate markers. Survival was not an analytical endpoint. Samples of tumours and serum were also snap‐frozen for analysis of drug concentrations using LC–MS/MS.

### Immunostaining

Paraffin‐embedded sections were dewaxed and rehydrated, and antigens were retrieved and immunostained using a range of antibodies (supplementary material, Table S2) as previously described 13, 16.

### Quantification

The quantification of all cell counts and intensity of staining in the organotypic sections was performed on four to six representative pictures per organotypic gel, of which there were three technical replicates for each biological replicate (minimum three). For the KPC mice, either the total tumour area or at least ten representative pictures per total tumour area were scanned using either an Axioplan microscope (Zeiss 40 V 4.8.10; Carl Zeiss MicroImaging, LLC, Thornwood, NY, USA), a confocal laser scanning microscope LSM 510 (Carl Zeiss MicroImaging, LLC) or a Pannoramic 250 High Throughput Scanner (3DHISTECH Ltd, Budapest, Hungary). The intensities of fluorescence in the green/red channels were normalized with IgG controls and background fluorescence and calculated in an unbiased, blinded manner using either Adobe Photoshop CS6 (Adobe Systems Incorporated, San Jose, CA, USA) or Pannoramic Viewer Software (3DHISTECH Ltd), and Image J software (NIH, MD, USA) as described before 13. The methods for measuring gel length and thickness, cancer, and stellate total cell number per gel are described elsewhere 11.

### Tissue gemcitabine and ATRA levels

Tissue samples were homogenized in 50% acetonitrile:water at a concentration of 100 mg/ml using a Precellys homogenizer. An aliquot of the homogenate was precipitated with acetonitrile containing a deuterium‐labelled internal standard of ATRA. Measurement was carried out against a calibration line prepared in mouse plasma homogenate (100 mg/ml) in 50% acetonitrile:water. The MS/MS used was a Sciex 4000Qtrap equipped with a heated nebulizer atmospheric pressure chemical ionization source operating in the negative mode at 350 °C. MRM transitions were 301–205 and 306–205 for unlabelled and labelled ATRA, respectively. LC was performed using a Dionex Ultimate 3000 LC and autosampler, using a gradient separation on a Phenomenex Kinetex 2.6 µm, 150 × 2.1 mm column. The binary gradient was run at 0.2 ml/min, starting at 40:60 A:B changing to 10:90 A:B from 0 to 15 min, then holding from 15 min to 17.6 min before quickly ramping back to 40:60 A:B at 17.62 min. The LC–MS/MS system was controlled by Analyst 1.4 software. In order to ensure that the correct isomer (ATRA) was measured, a system suitability test was run at the beginning and end of the sample analysis to demonstrate separation of the ATRA isomer from the 9‐ and 13‐*cis* isomers of retinoic acid (data not shown).

Fresh‐frozen tumour and plasma samples were processed and analysed for gemcitabine and its metabolites by LC–MS/MS as previously described 24.Briefly, LC–MS/MS was performed on a TSQ Vantage triple‐stage quadrupole mass spectrometer (Thermo Fisher Scientific, Waltham, MA, USA) fitted with a heated electrospray ionization (HESI‐II) probe operated in positive and negative mode at a spray voltage of 2.5 kV and a capillary temperature of 150 °C. Quantitative data acquisition was performed using LC Quan2.5.6 (Thermo Fisher Scientific).

### Statistical analysis

Statistical analysis and graphical data representation were performed using the software PRISM V.6 (GraphPad Software, Inc, La Jolla, CA, USA). Summary data are expressed as the median with interquartile range, since the distribution was non‐Gaussian. Comparisons were performed using the Kruskal–Wallis test with Dunn's multiple comparison test. The level of significance was set at *p* < 0.05.

## Results

Dosing schedule and timing for treatment of organotypic cultures and KPC mice with gemcitabine and ATRA were designed to mimic clinically relevant treatment regimens for advanced human pancreatic cancer, based on available data 3, 7, 11, 13, 22. *In vitro* optimizations such as growth inhibition to 50% of control (GI_50_) levels for gemcitabine were determined for translation into the organotypic 3D model. Interestingly, we found the presence of extracellular matrix (ECM) protein in the 3D model to have a preferential cytoprotective effect on the pancreatic stellate cells (PSCs). This resulted in different GI_50_ values for both PSCs and cancer cells in the 3D models compared with 2D culture (supplementary material, Figure S1; data not shown). Previously, we had demonstrated that ATRA had no direct effect on cancer cells by performing PSC or cancer cell alone organotypic cultures 13.

We then sought to identify effects on the cancer cells and stellate cells separately within this experimental design mimicking advanced PDAC. There was no change in gel contractility in organotypic cultures with any of the agents, compared with vehicle treatment (supplementary material, Figure S2).

**Figure 1:**
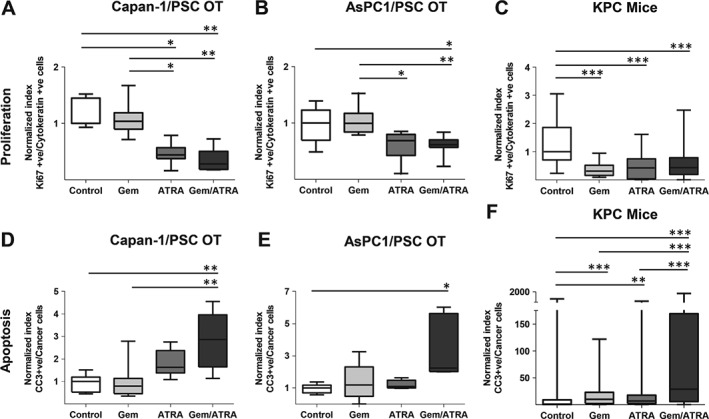
Cancer cell proliferation and apoptosis after combination treatment with gemcitabine and ATRA. Summary data from organotypic cultures (OT) and LSL‐Kras^G12D/+^;LSL‐Trp53^R172H/+^;Pdx‐1‐Cre mice (KPC mice) treated with either vehicle, gemcitabine alone, ATRA alone or a combination of gemcitabine with ATRA, as shown by median and interquartile range as box and whisker (min–max) plots. All observations were normalized to controls (vehicle). Nine to 15 experimental replicates were carried out for OT, resulting in 35–50 high‐power field measurements. Five to six mice per group were enrolled to allow assessments in 10–30 high‐power fields. Comparisons were made by the Kruskal–Wallis test followed by Dunn's post‐hoc analysis. ^***^p < 0.001; ^**^p < 0.01; ^*^p < 0.05. PSC = pancreatic stellate cell. (A–C) Cancer cell proliferation index in organotypics (A, B) and KPC mice (C). (D–F) Cancer cell apoptotic index in organotypics (D, E) and KPC mice (F). See supplementary material, Figure S3 for representative images.

**Figure 2:**
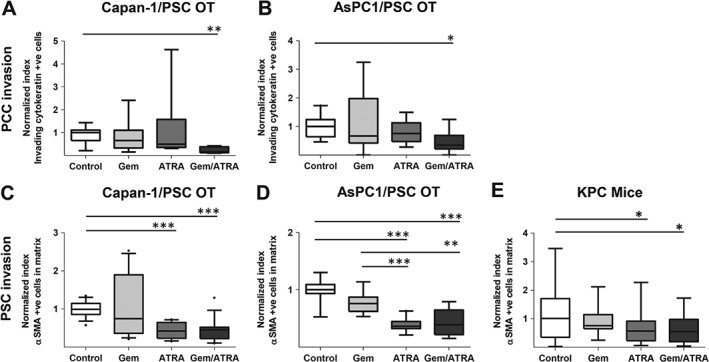
Invasion of cancer and stellate cells after combination treatment with gemcitabine and ATRA. Summary data from organotypic cultures (OT) and LSL‐Kras^G12D/+^;LSL‐Trp53^R172H/+^;Pdx‐1‐Cre mice (KPC mice) treated with either vehicle, gemcitabine alone, ATRA alone or a combination of gemcitabine with ATRA, as shown by median and interquartile range as box and whisker (min–max) plots. All observations were normalized to controls (vehicle). Nine to 15 experimental replicates were carried out for OT, resulting in 35–50 high‐power field measurements. Five to six mice per group were enrolled to allow assessments in ten high‐power fields. Comparisons were made by the Kruskal–Wallis test followed by Dunn's post‐hoc analysis. ^***^p < 0.001; ^**^p < 0.01; ^*^p < 0.05. PCC = pancreatic cancer cell; PSC = pancreatic stellate cell. (A, B) Cancer cell invasion index in organotypics. (C, D) Stellate cell invasion index in an organotypic model. (E) Stellate cell density in KPC mice. Stellate cell density in KPC was determined as green signal pixel intensity per area; the number of stellate cells was not counted, as it was not possible to identify accurately this cell type in the KPC tumour sections. See supplementary material, Figure S4 for representative images.

There was a significant reduction in proliferation of cancer cells induced by the presence of ATRA, either alone or in combination with gemcitabine, *in vivo* as well as *in vitro*, across all experimental conditions (Figures 1A–1C and supplementary material, Figures S3A and 3B). No significant difference was noted for stellate cell proliferation after any of the treatments (supplementary material, Figure S3C). However, induction of apoptosis was more pronounced with introduction of ATRA in the combination arm, suggesting that gemcitabine potentiates the effect of ATRA (Figures 1D–1F and supplementary material, Figures S3D and 3E). We found no significant difference in the apoptotic index of stellate cells after any of the treatments, in either of the PDAC models (supplementary material, Figure S3 F). Cancer cell invasion into the ECM, a surrogate marker for metastatic capability, was also reduced by the combination treatment (Figures 2A, 2B and supplementary material, Figures S4A, 4B).

**Figure 3:**
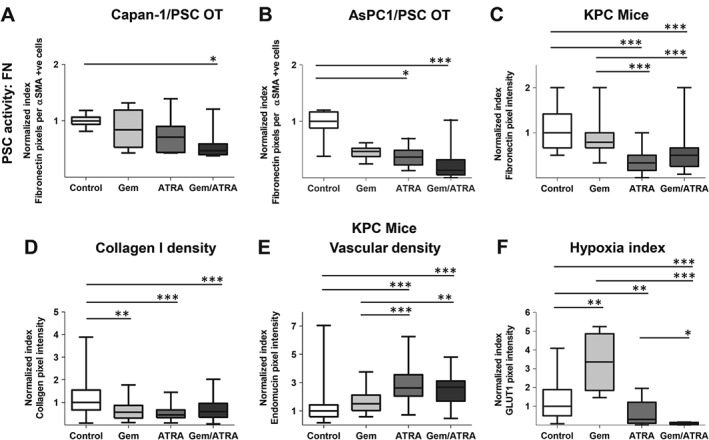
Pancreatic stellate cell activity, vascularity, and hypoxia after combination treatment with gemcitabine and ATRA. Summary data from organotypic cultures (OT) and LSL‐Kras^G12D/+^;LSL‐Trp53^R172H/+^;Pdx‐1‐Cre mice (KPC mice) treated with either vehicle, gemcitabine alone, ATRA alone or a combination of gemcitabine with ATRA, as shown by median and interquartile range as box and whisker (min–max) plots. All observations were normalized to controls (vehicle). Nine to 15 experimental replicates were carried out for organotypics, resulting in 35–50 high‐power field measurements. Five to six mice per group were enrolled to allow assessments in 10–30 high‐power fields. Comparisons were made by the Kruskal–Wallis test followed by Dunn's post‐hoc analysis. ^***^p < 0.001; ^**^p < 0.01; ^*^p < 0.05. PSC = pancreatic stellate cell. (A–D) Stellate cell activity in terms of fibronectin deposition in an organotypic model (A, B) and KPC mice (C) and in terms of collagen I deposition in the KPC mouse model (D). (E) Vascular density as determined by endomucin stain in the KPC mouse model. (F) Hypoxic index as determined by GLUT‐1 stain. See supplementary material, Figures S5 and 6 for representative images.

PSC invasion into the ECM and stellate cell density in mouse tumours were reduced by ATRA treatment alone, and in combination with gemcitabine (Figures 2C–2E and supplementary material, Figures S4A–4C). PSC numbers within organotypic gels did not change, reflecting the protective effect of matrix proteins on PSCs in 3D, not seen in the 2D *in vitro* state (supplementary material, Figures S1B, 2D, and 2E). However, the PSC activation state was altered by ATRA and the combination of gemcitabine and ATRA, as indicated by a significant reduction in deposition of ECM substrates such as fibronectin and collagen I, implying stromal remodelling (Figures 3A–3D and supplementary material, Figures S5A–5C).

**Figure 4:**
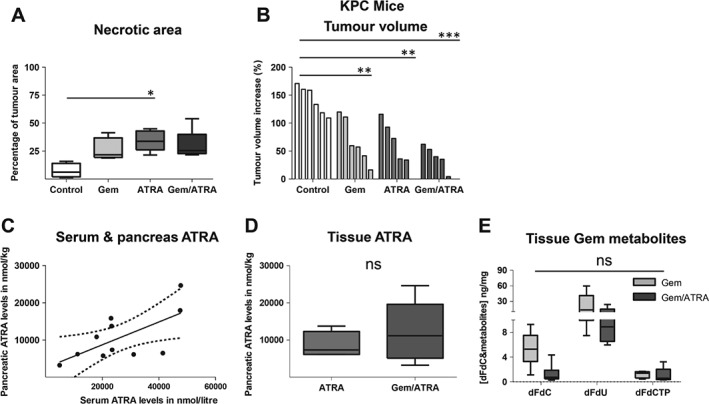
Effect of combination treatment with gemcitabine and ATRA on tumour growth and gemcitabine and ATRA intra‐tumoural levels in KPC mice. (A) Percentage necrotic area as determined by H&E staining. Summary data from LSL‐Kras^G12D/+^;LSL‐Trp53^R172H/+^;Pdx‐1‐Cre mice (KPC mice) treated with either vehicle, gemcitabine alone, ATRA alone or a combination of gemcitabine with ATRA, as shown by median and interquartile range as box and whisker (min–max) plots. Five to six mice per group were enrolled. Comparisons were made by the Kruskal–Wallis test followed by Dunn's post‐hoc analysis. ^***^p < 0.001; ^**^p < 0.01; ^*^p < 0.05. See supplementary material, Figure S6C for representative images. (B) Percentage change in tumour volume between pre‐treatment (day −2) and post‐treatment (day 7) was measured by ultrasound in the KPC mouse model. (C) Serum and pancreatic tumour ATRA concentration demonstrated a correlation in mice receiving ATRA treatment [Pearson's correlation coefficient 0.66 (95% CI 0.09–0.9)]. A regression line and its 95% confidence interval are shown. (D) Tumour tissue ATRA concentration in KPC mice treated with ATRA or gemcitabine/ATRA. (E) Tumour tissue gemcitabine metabolites in gemcitabine‐ and gemcitabine/ATRA‐treated mice. ns = not significant.

Together with stromal remodelling, we demonstrated increased vascularity of the KPC tumours, associated with decreasing hypoxia (Figures 3E, 3 F and supplementary material, Figures S6A, 6B). Surprisingly, despite this reduction in hypoxia, there was increased necrosis, *in vivo*, with combination treatment (Figure 4A and supplementary material, Figure S6C). This resulted in smaller tumours in mice treated with combination therapy (Figure 4B). Certainly, with this regimen, both agents can be delivered successfully *in vivo* into the tumour parenchyma, as measured by LC–MS/MS (Figures 4C–4E). Furthermore, the tissue ATRA (not 9‐*cis* and 13‐*cis* RA) is directly correlated to serum ATRA measurements, allowing surrogate measurements to be easily and readily performed (Figure 4C).

The precise mechanism(s) underpinning the success of this combination therapy are difficult to pinpoint, since ATRA influences multiple signalling cascades 13. The enhanced apoptosis and reduction in proliferation of cancer cells may result from the reduction of Wnt signalling in the tumour compartment 13, disrupted fibroblast growth factor (FGF) signalling in the stromal compartment 17, or targeting of other signalling cascades such as hedgehog, IL6, and CXCL12 14.

**Figure 5:**
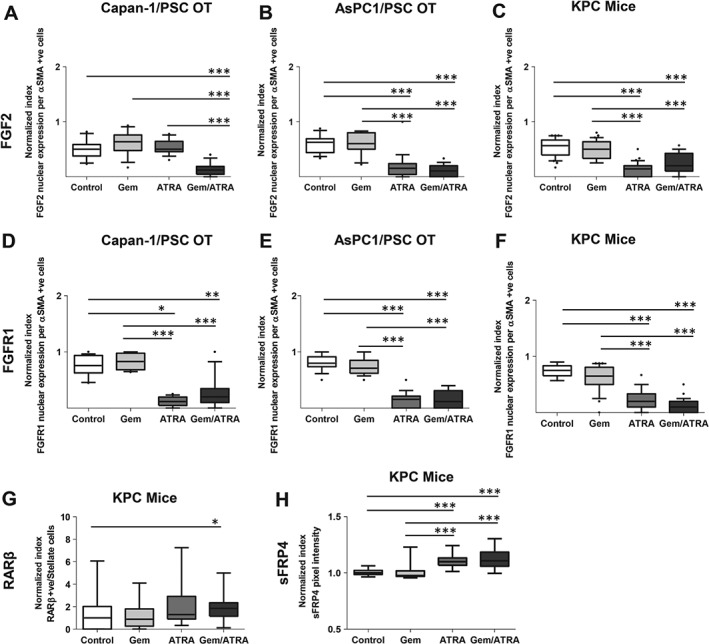
The combination of gemcitabine with ATRA affects multiple signalling cascades in cancer cells and stroma in organotypic cultures and KPC mice. Summary data from organotypic cultures (OT) and LSL‐Kras^G12D/+^;LSL‐Trp53^R172H/+^;Pdx‐1‐Cre mice (KPC mice) treated with either vehicle, gemcitabine alone, ATRA alone or a combination of gemcitabine with ATRA, as shown by median and interquartile range as box and whisker (min–max) plots. All observations were normalized to controls (vehicle). Sections from three experimental replicates were carried out for organotypics, resulting in 18 high‐power field measurements. Three mice per group were selected to allow assessments in ten high‐power fields per section. Comparisons were made by the Kruskal–Wallis test followed by Dunn's post‐hoc analysis. ^***^p < 0.001; ^*^p < 0.05. (A–C) FGF2 nuclear expression index in organotypics and KPC mice. (D–F) FGFR1 nuclear expression index in organotypics and KPC mice. (G) RARβ nuclear expression index in KPC mice. (H) sFRP4 stromal expression index in KPC mice. See supplementary material, Figures S7–9 for representative images.

**Figure 6:**
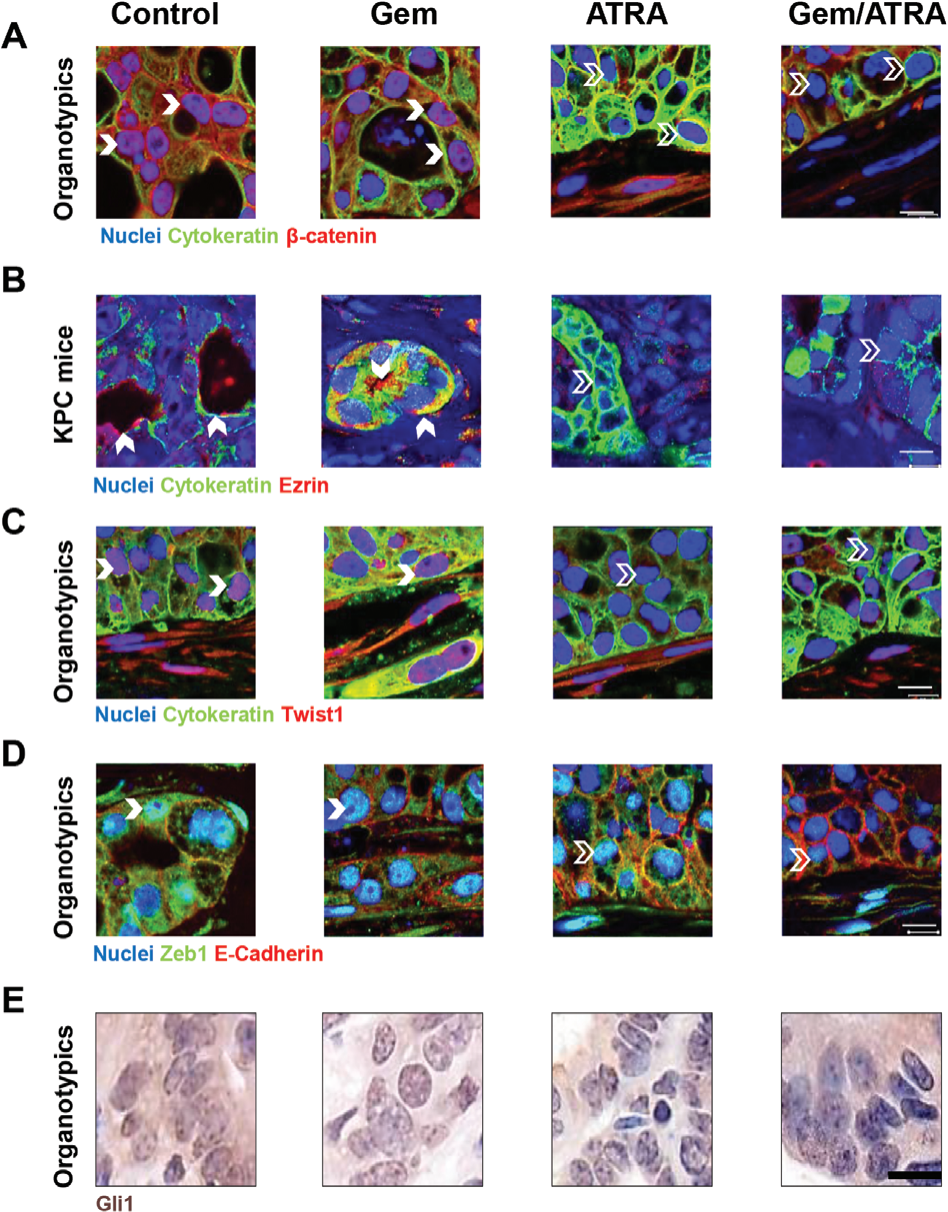
The combination of gemcitabine with ATRA affects apical polarity, epithelial–mesenchymal transition, and hedgehog signalling in cancer cells within organotypic cultures and KPC mice. Representative images from organotypic cultures (OT) and LSL‐Kras^G12D/+^;LSL‐Trp53^R172H/+^;Pdx‐1‐Cre mice (KPC mice), as indicated, treated with either vehicle, gemcitabine alone, ATRA alone or the combination of gemcitabine with ATRA. Bold arrowheads indicate positive stain and other arrowheads indicate negative stain. (A) Capan‐1 cells stained with anti‐cytokeratin (green) and anti‐β‐catenin (red) antibodies were used to localize β‐catenin in organotypic cultures. Cytokeratin‐positive cancer cells demonstrate loss of nuclear β‐catenin in ATRA‐treated organotypic cultures. See supplementary material, Figure S10 for detailed data on KPC mice and organotypic cultures. Scale bar = 10 µm. (B) Anti‐cytokeratin (green) and anti‐ezrin (red) antibodies were used to localize ezrin in KPC mice. Cytokeratin‐positive cancer cells demonstrate loss of membranous ezrin in ATRA‐treated murine tissues. See supplementary material, Figure S11 for detailed data on KPC mice and organotypic cultures. (C**)** Anti‐cytokeratin (green) and anti‐TWIST1 (red) antibodies were used to localize TWIST1 in Capan‐1/PS1 organotypic cultures. Cytokeratin‐positive cancer cells demonstrate loss of nuclear TWIST1 in ATRA organotypic cultures. Cytokeratin‐negative PSCs demonstrate nuclear TWIST1 to act as an internal positive control. See supplementary material, Figure S12 for detailed data on KPC mice and organotypic cultures. (D**)** Anti‐ZEB1 (green) and anti‐E‐cadherin (red) antibodies were used to localize ZEB1 in Capan‐1/PS1 organotypic cultures. E‐cadherin‐positive cancer cells demonstrate loss of nuclear ZEB1 in ATRA organotypic cultures. E‐cadherin‐negative PSCs demonstrate nuclear ZEB1 to act as an internal positive control. See supplementary material, Figure S13 for detailed data on KPC mice and organotypic cultures. (E**)** In KPC mice, anti‐Gli1 staining (brown) was used to localize Gli1 expression. Loss of nuclear Gli1 in epithelial‐appearing cells was demonstrable within ATRA‐treated murine PDAC tissues. See supplementary material, Figure S14 for detailed data on KPC mice. Scale bar = 10 µm.

In our experimental models, we could demonstrate a reduction in nuclear translocation of FGF2 and FGFR1 in PSCs upon treatment of KPC mice and organotypic cultures with ATRA (Figures 5A–5F and supplementary material, Figures S7 and 8), this co‐translocation being pertinent to PSC activity, as demonstrated before 13. There was enhanced nuclear retinoic acid receptor β (RARβ) visibility within the PSCs in KPC tumours upon treatment with ATRA (Figure 5G and supplementary material, Figure S9A), which is known to reflect PSC quiescence 13. Up‐regulation of PSC RARβ activity enhances the secretion of secreted Frizzled related protein 4 (sFRP4) from the quiescent PSCs, as demonstrated before 13. We could demonstrate increased stromal sFRP4 upon treatment with ATRA (Figure 5H and supplementary material, Figure S9B). In turn, this modulation within PSCs upon treatment with ATRA led to reduced nuclear β‐catenin, which translocates to the nucleus following canonical Wnt signalling activation 13, 25 (Figure 6A and supplementary material, Figure S10). sFRP4 can sequester Wnt ligands within stroma to abrogate canonical Wnt signalling flux, and also act as a gatekeeper, and thus affect epithelial–mesenchymal transition (EMT), apoptosis, and invasion within cancer cells 26, 27.

In addition, there was a reduction in ezrin expression (Figure 6B and supplementary material, Figure S11), which has previously been shown to enhance podosomal rosette formation 25. Ezrin is also a marker for lumen formation and apico‐basal polarity, and such changes could be observed more clearly in the organotypic cultures than in KPC mice, but were difficult to quantify (data not shown). Furthermore, there was suppressed expression of EMT‐activating transcription factors, as evidenced by a reduction of nuclear *TWIST1* and *ZEB1* in PDAC cells (Figures 6C and 6D and supplementary material, Figures S12 and 13), which may be related to a reduction of canonical Wnt signalling 28. Lastly, there was also a reduction of nuclear and cytoplasmic Gli1 in cancer cells, suggesting reduced hedgehog signalling in cancer cells 29 (Figure 6E and supplementary material, Figure S14). The alteration of the vascular and immune sub‐compartments of stroma, as shown by us before 14, 30, could also lead to enhanced necrosis in tumours *in vivo*, especially with combination treatment, ultimately resulting in tumour shrinkage.

## Discussion

The reduced tumour growth with a combination of stromal and cancer cell co‐targeting could be clinically relevant, since many locally advanced and borderline resectable cancers may be rendered surgically resectable using this regimen, a hypothesis which is now ready to be tested in clinical trials. The findings reported here contrast with two recent studies exploring more radical approaches involving complete stromal ablation 8, 9 suggesting that stromal ‘normalization’ is much preferred over stromal ablation approaches 10. Rhim *et al* recently demonstrated that ablating sonic hedgehog‐dependent stroma resulted in a more vascular tumour with poor differentiation, which, in part, could be abrogated by VEGF signalling blockade 8. In a parallel approach, Ozdemir *et al*, by genetically ablating α‐SMA‐positive stroma, demonstrated the presence of more invasive tumours, characterized by hypoxia, an epithelial‐to‐mesenchymal transition, and alterations in immune surveillance. Specifically, this resulted in increased CD4^+^Foxp3^+^ T‐regulatory cell infiltration, leading to a more aggressive tumour phenotype 8, 9. In contrast, our findings suggest that restoring homeostatic stromal characteristics, rather than stromal ablation, has a tumour‐suppressive rather than a tumour‐enhancing effect. This may be due in part to the homeostatic role of naturally occurring vitamin A analogue, and in part to the pleiotropic actions of ATRA, which are of relevance to pancreatic embryology and development.

Indeed, it has been demonstrated that ATRA influences multiple signalling cascades through selective retinoid receptor signalling (retinoid versus rexinoid receptors and isoforms of both subsets such as α, β, γ) in embryonic pancreas development, injury, and regeneration 13, 31, 32, 33, 34. In particular, ATRA has a predominant effect on acinar morphology rather than endocrine cells, due to the epithelial–mesenchymal interactions in the developing pancreas 34. Retinoic acid is critical for the developing pancreas, where it can interact with, and influence, Wnt, TGFβ (transforming growth factor β), BMP (bone morphogenetic protein), and other signalling cascades 35, all of which are understood to be hijacked and altered by cancer cells to recruit stromal cells 36. Previously, we had demonstrated that restoring the quiescent nature of PSCs using ATRA can alter the signalling flux within the tumour–stroma 13 as well as intra‐stromal cross‐talk 14 of key pathways relevant to pancreatic cancer progression.

The enhanced apoptosis and reduction in proliferation of cancer cells seen in this study may result from a reduction of canonical Wnt signalling in the tumour compartment, as a result of modification in the stromal compartment, by sequestering Wnt ligands, due to sFRP4 secretion 13. Furthermore, the disrupted fibroblast growth factor (FGF) signalling in the stromal compartment 17, or targeting of other signalling cascades such as hedgehog, IL6, or CXCL12 14, could detrimentally affect the cancer cells by altering the signalling flux of (rather than selectively ablating) key cascades. FGF2/FGFR1 nuclear translocation is vital to activation of PSCs, which is required for cancer progression 17. Other modifications in ECM deposition and remodelling such as collagen and fibronectin can affect the cyto‐protective micro‐environment of cancer cells 37, internalization and recycling of key integrins 18, and migration/invasion of cancer cells 38 as well as immune cells 14.

This alteration of a number of signalling pathways, in turn, may affect key epithelial cellular processes such apico‐basal polarity 25, matrix remodelling 18, and epithelial–mesenchymal transition 26, and thus halt cancer progression. Therefore, multiple tumour–stroma cross‐talk signalling cascades affecting numerous cancer and stellate cell processes are altered by ATRA when administered in a clinically achievable dosing schedule.

Moreover, the alteration of immune cell infiltrate, vascularity, and hypoxia demonstrated by us previously 13, 14, 30 in relation to stromal targeting therapy is relevant to this combination treatment. We demonstrate here that chemotherapy is more effective when combined with ATRA in altering intra‐stromal or peri‐tumoural cross‐talk in the tissue micro‐environment, particularly the enhancement of vascularity and the consequent reduction in hypoxia. In fact, it has long been understood that the tumour micro‐environment exerts a protective influence on cancer cells through multiple mechanisms such as resistance to alkylating agents 39 and direct cell–cell contact between cancer and stromal cells 40, through many signalling cascades, thus enhancing the tumour cell autonomous resistance to chemotherapy. We were unable to detect enhanced levels of the active metabolite of gemcitabine, and we speculate that this may be related to an increased necrotic component of the tumour, which will not contain active metabolite. In addition, the consistency of the results obtained from these two different PDAC models (one *in vitro*, one *in vivo*) suggests that the organotypic models may be useful preclinical tools for dissection of the molecular signalling pathways involved in PDAC drug resistance. Modulating the 3D OT cultures would recapture important aspects of the tumour micro‐environment that can influence cancer cell behaviour.

Thus, based on the data presented here, we postulate that the effect of this combination strategy of co‐targeting cancer and stromal cells is more likely to involve dampening of a multitude of signalling cascades, rather than via a single, specific pathway or mechanism, and therefore altering a number of key cellular processes. This notion concurs with separate attempts to restore the homeostatic capability of desmoplastic stroma by targeting the vitamin D receptor 15, as well as normalization of vascularization with dual‐action combination therapy 30. Given the known fat‐soluble vitamin deficiency in patients with PDAC, due to biliary and pancreatic duct obstruction, the proposal to restore homeostatic stromal function, in conjunction with cancer targeting with conventional chemotherapy, appears to be a viable therapeutic opportunity underpinned by the clinical features of this cancer. This hypothesis, therefore, has enough rationale to be explored in a human clinical trial involving patients with PDAC.

**Abbreviations**
ATRA: all‐*trans* retinoic acid
BMP: bone morphogenetic protein
FBS: fetal bovine serum
FGF: fibroblast growth factor
FGFR: fibroblast growth factor receptor
KPC: *LSL‐Kras^G12D/+^;LSL‐Trp53^R172H/+^;Pdx‐1‐Cre*
LC–MS: liquid chromatography–mass spectrometry
PDAC: pancreatic ductal adenocarcinoma
PSC: pancreatic stellate cells
RA: retinoic acid
RARβ: retinoic acid receptor β
SHH: sonic hedgehog
STR: short tandem repeat
TGFβ: transforming growth factor β

## Author contribution statement

The authors contributed in the following way: study concept, design and supervision, and obtained funding: HMK; acquisition of data: EFC, EG, CF, MDW, TEB, ASW, FRD, PA, and HMK; statistical analysis: EFC and HMK; drafting of the manuscript: HMK and EFC; technical or material support: RPG, FMR, NRL, and HMK; analysis and interpretation of data as well as critical revision of the manuscript for important intellectual content: all authors.

**SUPPORTING INFORMATION ON THE INTERNET**

The following supporting information may be found in the online version of this article:

**Figure S1.** Design of experiments and determination of dosing schedule.

**Figure S2.** The combination treatment of gemcitabine with ATRA does not affect PSC number, and consequently gel length and thickness are also unchanged.

**Figure S3.** The combination of gemcitabine with ATRA affects cancer cell proliferation and apoptosis in organotypic cultures, as well as in KPC mice.

**Figure S4.** The combination of gemcitabine with ATRA affects cancer and stellate cell invasion in organotypic cultures as well as stellate cell density in KPC mice.

**Figure S5.** ATRA alters stellate cells' activation status.

**Figure S6.** The combination treatment of gemcitabine with ATRA alters the vascular density, hypoxic environment, and the necrosis pattern in murine tumours.

**Figure S7.** ATRA treatment affects pancreatic stellate cell activity by reducing the nuclear translocation FGF2.

**Figure S8.** ATRA treatment affects the pancreatic stellate cell activity by reducing the nuclear translocation of FGFR1.

**Figure S9.** Nuclear RARβ expression in, and stromal sFRP4 secretion by, pancreatic stellate cells is altered upon treatment with ATRA alone and in combination with gemcitabine.

**Figure S10.** ATRA disrupts the Wnt–β‐catenin signalling pathway.

**Figure S11.** The combination treatment affects the lumen formation and apico‐basal polarity of cancer cells.

**Figure S12.** ATRA alone or in combination with gemcitabine affects nuclear *TWIST1* expression within cancer cells.

**Figure S13.** The combination treatment affects the nuclear translocation of transcription factor *ZEB1* in cancer cells.

**Figure S14.** The combination treatment affects the hedgehog signalling in cancer cells.

**Table S1.** KPC mice characteristics at recruitment.

**Table S2.** Table of antibodies.

## Supporting information


**Figure S1.** Design of experiments and determination of dosing schedule.

**Figure S2.** The combination treatment of gemcitabine with ATRA does not affect PSC number, and consequently gel length and thickness are also unchanged.

**Figure S3.** The combination of gemcitabine with ATRA affects cancer cell proliferation and apoptosis in organotypic cultures, as well as in KPC mice.

**Figure S4.** The combination of gemcitabine with ATRA affects cancer and stellate cell invasion in organotypic cultures as well as stellate cell density in KPC mice.

**Figure S5.** ATRA alters stellate cells' activation status.

**Figure S6.** The combination treatment of gemcitabine with ATRA alters the vascular density, hypoxic environment, and the necrosis pattern in murine tumours

**Figure S7.** ATRA treatment affects pancreatic stellate cell activity by reducing the nuclear translocation FGF2.

**Figure S8.** ATRA treatment affects the pancreatic stellate cell activity by reducing the nuclear translocation of FGFR1.

**Figure S9.** Nuclear RARβ expression in, and stromal sFRP4 secretion by, pancreatic stellate cells is altered upon treatment with ATRA alone and in combination with gemcitabine.

**Figure S10.** ATRA disrupts the Wnt–β‐catenin signalling pathway.

**Figure S11.** The combination treatment affects the lumen formation and apico‐basal polarity of cancer cells

**Figure S12.** ATRA alone or in combination with gemcitabine nuclear Twist1 expression within cancer cells.

**Figure S13.** The combination treatment affects the nuclear translocation of transcription factor ZEB1 in cancer cells.

**Figure S14.** The combination treatment affects the hedgehog signalling in cancer cells.

**Table S1.** KPC mice characteristics at recruitment.
**Table S2.** Table of antibodies.
