# Supplementary material for: Anti‐stromal treatment together with chemotherapy targets multiple signalling pathways in pancreatic adenocarcinoma
Source: J Pathol. 2016 May 25;239(3):286–96. doi: 10.1002/path.4727 (PMC5025731; doi:10.1002/path.4727)
Supplement: Supplementary file 15 — Table S1. KPC mice characteristics at recruitment. Table S2. Table of antibodies. [file PATH-239-286-s014.doc]

**SUPPORTING INFORMATION**

**Supplementary figure legends**

**Supplementary Figure 1. Design of experiments and determination of dosing schedule.**

A) Determination of Gemcitabine GI_50_ (Cytotoxic effect: growth inhibition of 50%) alone, or in combination with ATRA (1μM), on AsPC1 (blue and black lines) or Capan-1 (green and red lines) cell growth. Pancreatic cancer cell lines AsPC-1 and Capan-1 in 2D monoculture were exposed once, to different concentrations of Gemcitabine, and were allowed to grow for a period of up to seven days, prior to assessing proliferation rates. The GI_50_ for Gemcitabine was determined as 240nM for AsPC1, and 48nM for Capan-1, indicating that AsPC1 cells are more resistant to Gemcitabine, when compared to Capan-1 cell line. Addition of daily ATRA, to this treatment regimen had no effect on the GI_50_ curves of either cell line, indicating the lack of any combinational effect of ATRA with Gemcitabine in 2D monocultures on cancer cells.

B) Determination of Gemcitabine GI_50_ alone or in combination with ATRA (1μM) on pancreatic stellate cells (PSC) growth. PSC showed sensitivity to Gemcitabine (GI_50_ 26nM), which increased when treatment was combined with daily ATRA exposure. At least, in 2D cultures, the actively proliferating PSC appear to be sensitive to Gemcitabine.

C) i) Treatment protocol of the 3D organotypic cultures with Gemcitabine weekly for two consecutive weeks, mimicking treatment currently in use in the clinic [3]. Representative images of H&E stained sections of gels resultant from AsPC1 (ii) or Capan-1 (iii) organotypic cultures, treated with Gemcitabine at various doses in order to determine Gemcitabine GI_50_ (concentration that reduces epithelial cell layer thickness by 50%). Gemcitabine GI_50_ was slightly higher at 300nM for AsPC1 and 100nM for Capan-1 organotypic cultures than in the 2D monocultures. The increased value of GI_50_ is anticipated due to cyto-protective effect of organized 3D matrix particularly Collagen I [41]. Intriguingly PSC layer thickness was unaffected by Gemcitabine treatment, when PSC and cancer cells were combined (data not shown).

D) Representative image of a section of an organotypic culture treated with BrdU (red) and stained with a cytokeratin antibody (green) to delineate cancer cells. Representative graph of the percentage of cancer and stellate cells with BrdU incorporation. To determine the rates of nucleoside uptake in organotypic cultures, BrdU pulse chase was carried out. BrdU is an analogue of the nucleoside thymidine, and Gemcitabine an analogue of cytidine. BrdU was administered at the same concentrations that Gemcitabine would be added to the 3D co-culture models. The percentage of incorporation of BrdU by PSC was much less than by tumour cells, which confirms that Gemcitabine has minimal cytotoxic effect on PSC in organotypic culture model. This effect may be due to a slower proliferation rate of PSC. Thus, they do not incorporate the nucleoside analogue at the same rate as cancer cells. Therefore, the cytotoxic effect of Gemcitabine is largely specific to the epithelial cancer cells.

**Supplementary Figure** **2.** **The combination treatment of gemcitabine with ATRA does not affect PSC number, and consequently gel length and thickness are also unchanged.**

**A)** Schematic representation of an organotypic culture of admixed cancer cells and PSC, seeded on top of gel composed of Matrigel and Collagen I that mimics the tumour ECM environment. Measurements of cancer cell layer thickness, gel length, gel thickness are schematically represented and have been previously described [11].

**B, C)** Cancer cell layer thickness was unaffected in presence of PSC in Capan-1/PS1 (B) and AsPC1/PS1 (C) organotypic cultures, respectively, upon treatment with vehicle, Gemcitabine alone, ATRA alone or a combination of Gemcitabine and ATRA.

**D,E)** Total PSC number was also unaffected in Capan-1/PS1 (D) and AsPC1/PS1 (E) organotypic cultures respectively upon treatment.

**F,G)** Gel thickness was also unaffected in Capan-1/PS1 (F) and AsPC1/PS1 (G) organotypic cultures, respectively, upon treatment.

**H,I)** Gel length was also unaffected in Capan-1/PS1 (H) and AsPC1/PS1 (I) organotypic cultures, respectively, upon treatment.

9-15 experimental replicates were carried out for organotypic cultures. Comparisons were made by Kruskal-Wallis test followed by Dunn’s post-hoc analysis.

ns: not significant

**Supplementary Figure 3.** **The combination of gemcitabine with ATRA affects cancer cell proliferation and apoptosis in organotypic cultures, as well as in KPC mice.**

A) Representative images from organotypic cultures (OT) treated with either vehicle, Gemcitabine alone, ATRA alone or the combination of Gemcitabine with ATRA. Capan-1 cells stained with an anti-cytokeratin antibody (green) and proliferating Capan-1 cells stained with an anti-Ki67 antibody (red) to determine ratio of proliferating cancer cells per field.

B) Representative images of tumour sections from *LSL-Kras^G12D/+^;LSL-Trp53^R172H/+^;Pdx-1-Cre* mice (KPC mice) treated with vehicle, Gemcitabine, ATRA or Gemcitabine with ATRA, and stained with an anti-CK8 antibody (red) and proliferating cancer cells stained with an anti-Ki67 antibody (green) to determine the ratio of proliferating cancer cells per field.

C) Representative images from organotypic cultures (OT) after same treatment regimen, where Stellate cells were stained with an anti-alpha-SMA antibody in green and with an anti-Ki67 antibody (red) to determine ratio of proliferating stellate cells per field.

D) Representative images of organotypic gel sections where Capan-1 cells were stained by immuno-histochemistry with an anti-cleaved caspase-3 antibody. Apoptotic cancer cells were identified by the cytoplasmic brown staining. There was no staining within the PSC layer. Percentage apoptotic cancer cells (based on morphology) were determined.

E) Representative images of tumour sections from KPC mice stained with an anti-cleaved caspase-3 antibody to determine apoptotic cells. There was no staining in non-epithelial compartment. Percentage apoptotic cancer cells (based on morphology) were determined.

F) Representative images of tumour sections from KPC mice stained by immunofluorescence with an anti-alpha-SMA antibody and with an anti-cleaved caspase-3 antibody to determine the ratio of apoptotic stellate cells. There was no staining in the stromal compartment.

**Supplementary Figure 4.** **The combination of gemcitabine with ATRA affects cancer and stellate cell invasion in organotypic cultures as well as stellate cell density in KPC mice.**

**A)** Representative images of organotypic gel sections where Capan-1 cells were stained with a cytokeratin antibody (green) and PSC were stained with an anti-αSMA antibody (red) to identify the cells that have invaded the gel. The yellow line marks the junction between the PSC layer and the extracellular matrix (top of the gel). The number of invading cells was counted directly on the section on the Axioplan microscope, to accurately identify the top of the gel and identify the cell type and number that invaded into the gel.

**B)** i and ii) Representative H&E stained image from a Capan-1/PS1 OT section that clearly shows the cancer cell layer, the gel and the top of the gel where the stellate cell layer is demonstrable. The dashed black line in ii) marks the top of the gel. Invading cells were counted below the black line.

C) Representative images of tumour sections from KPC mice stained with an anti-αSMA antibody (green) to identify PSC. Stromal cell density was determined by green pixel intensity. Pericytes were accounted for as described in Figure 6A. Scale bar 100 µm (except C where Scale bar = 50 µm).

Supplementary Figure 5. ATRA alters stellate cells’ activation status.

A) Representative images of organotypic sections where Capan-1 cells were stained with an anti-Cytokeratin antibody (green) and extracellular matrix (ECM) deposition by an anti-Fibronectin antibody (red). Note: the ECM gel formed at inception with Collagen I and Matrigel contains no fibronectin. Hence, fibronectin shown here represents ECM generated by cells co-cultured in 3D. Fibronectin deposition was only present around PSC, indicating PSC were source of this ECM protein. Scale bar 100 µm.

B) Representative images of tumour sections from *LSL-Kras^G12D/+^;LSL-Trp53^R172H/+^;Pdx-1-Cre* mice (KPC mice) stained by immuno-histochemistry with anti-Fibronectin antibody. Fibronectin expression was scored based on intensity and degree of brown staining as described before [14]. Scale bar 50 µm.

C) Representative images of Collagen deposition in KPC mice tumour sections stained with Picrosirius Red. Pixel intensity was determined as described before [30]. Scale bar 100 µm.

Supplementary Figure 6. The combination treatment of gemcitabine with ATRA alters the vascular density, hypoxic environment, and the necrosis pattern in murine tumours.

A) Representative images of tumour sections from *LSL-Kras^G12D/+^;LSL-Trp53^R172H/+^;Pdx-1-Cre* mice (KPC mice) stained with an anti-Endomucin antibody (red) to identify blood vessels and an anti-αSMA antibody (green) to identify stellate cells as well as pericytes. The number of blood vessels increased in the tumour/stromal area of PDAC tumours of mice treated with ATRA or with the combination Gemcitabine /ATRA, while at the same time there is a reduction of αSMA expression (after subtracting doubly stained structures to exclude pericytes). Scale bar 100 µm.

B) Representative images KPC mice tumour sections stained with an anti-GLUT1 antibody (green) to mark hypoxic areas in the tumours. Pixel intensity determined level of hypoxia as described before. Scale bar 100 µm.

C) Representative images of H&E stained tumour sections from mice. Necrotic areas, identified by morphology, are marked by the dotted lines in black. Percentage of necrotic area was determined based on total surface area of tumour. Scale bar 5000 µm.

Supplementary Figure 7. ATRA treatment affects pancreatic stellate cell activity by reducing the nuclear translocation FGF2.

A-D) Representative images of sections from *LSL-Kras^G12D/+^;LSL-Trp53^R172H/+^;Pdx-1-Cre* mice (KPC mice) stained with an anti-FGF2 antibody (red) and an anti-Cytokeratin antibody (green) to identify epithelial cells.

E-H) Representative images of Capan-1/PS1 organotypic (OT) sections stained with same antibodies as KPC mice. There is a clear reduction of nuclear FGF2 expression in stromal cells (Cytokeratin –ve cells) from mice treated with ATRA. Scale bar 50 µm.

a-d) Zoom in images of the marked areas of KPC main images (A-D) with bold arrowheads pointing to nuclear FGF2 expressing stromal cells and empty arrowheads pointing to stromal cells not expressing FGF2 in the nucleus.

e-h) Zoom in images of the marked areas of OT main images (E-H) also with bold and empty arrowheads pointing to the difference of nuclear FGF2 expression in stellate cells upon treatment with ATRA. Scale bar 10 µm.

Supplementary Figure 8. ATRA treatment affects the pancreatic stellate cell activity by reducing the nuclear translocation of FGFR1.

A-D) Representative images of sections from *LSL-Kras^G12D/+^;LSL-Trp53^R172H/+^;Pdx-1-Cre* mice (KPC mice) stained with an anti-FGFR1 antibody (red) and an anti-αSMA antibody (green) to identify stromal cells.

E-H) Representative images of Capan-1/PS1 organotypic (OT) sections stained with same antibodies as KPC mice. There is a clear reduction of nuclear FGFR1 expression in stromal αSMA-positive cells in the mice treated with ATRA. Scale bar 50 µm.

a-d) Zoom in images of the marked areas of KPC main images (A-D) with bold arrowheads pointing to nuclear FGFR1 expressing stromal cells and empty arrowheads pointing to stromal cells with no nuclear FGFR1 expression.

e-h) Zoom in images of the marked areas of OT main images (E-H) also with bold and empty arrowheads pointing to the difference of nuclear FGFR1 expression in stellate cells upon treatment with ATRA which is in concordance with FGF2 expression pattern seen in Supplementary figure 7. Scale bar 10 µm.

Supplementary Figure 9. Nuclear RARβ expression in, and stromal sFRP4 secretion by, pancreatic stellate cells is altered upon treatment with ATRA alone and in combination with gemcitabine.

**A)** Representative images of tumour sections from *LSL-Kras^G12D/+^;LSL-Trp53^R172H/+^;Pdx-1-Cre* mice (KPC mice) stained by immuno-histochemistry with an anti-RARβ antibody. Nuclear RARβ expression is most enhanced in stellate cells of sections from mice treated with ATRA. Zoom in images show the amplification of the marked areas of main images, which show the nuclear RARβ expression in stetalle cells of sections from ATRA treated mice in comparison to Vehicle or Gemcitabine alone treated mice. Scale bar 100 µm. Zoom in images: Scale bar 10 µm.

**B)** Representative images of tumour sections from KPC mice stained by immuno-histochemistry with an anti-sFRP4 antibody. Stromal sFRP4 expression is most enhanced in tumour surrounding environment of sections from mice treated with ATRA alone or in combination with Gemcitabine. Scale bar 50 µm. Zoom in images show a significant expression of sFRP4 in the stroma of tumour from ATRA or ATRA/Gemcitabine combination treated mice in comparison to Vehicle or Gemcitabine alone treated mice. Zoom in images: Scale bar 10 µm.

Supplementary Figure 10. ATRA disrupts the Wnt–β-catenin signalling pathway.

**A-D)** Representative images of tumour sections from *LSL-Kras^G12D/+^;LSL-Trp53^R172H/+^;Pdx-1-Cre* mice (KPC mice) stained with an anti-β-catenin antibody (red) and an anti-Cytokeratin antibody (green) to identify epithelial cells.

**E-H)** Representative images of Capan-1/PS1 organotypic (OT) sections stained with same antibodies as KPC mice sections. Scale bar 50 µm.

**a-d)** Zoom in images of the marked areas of KPC main images (A-D) with bold arrowheads pointing to nuclear β-catenin expression in epithelial cells and empty arrowheads pointing to no nuclear β-catenin expression.

**e-h)** Zoom in images of marked areas of OT main images (E-H) also with bold and empty arrowheads pointing to the differences of nuclear β-catenin expression in epithelial cells. There is a shift of the spatial β-catenin localization that spans from the cell nuclei, in epithelial cell either from KPC tumours or OT cultures treated with vehicle or Gemcitabine alone to the cell membrane upon treatment with ATRA or Gemcitabine and ATRA. Scale bar 10 µm.

**Supplementary Figure 11. The combination treatment affects the lumen formation and apico-basal polarity of cancer cells.**

**A-D)** Representative images of tumour sections from *LSL-Kras^G12D/+^;LSL-Trp53^R172H/+^;Pdx-1-Cre* mice (KPC mice) stained with an anti-Ezrin antibody (red) and an anti-Cytokeratin antibody (green) to identify epithelial cells.

**E-H)** Representative images of Capan-1/PS1 organotypic (OT) sections stained with same antibodies as KPC mice sections. Scale bar 50 µm.

**a-d)** Zoom in images of the marked areas of KPC main images (A-D) with bold arrowheads pointing to Ezrin cell membrane expression in cancer cells and empty arrowheads pointing to loss of membranous Ezrin expression.

**e-h)** Zoom in images of the marked areas of OT main images (E-H) also with bold and empty arrowheads pointing to the differences in Ezrin expression in cancer cells. Ezrin expression is reduced in cancer cells of KPC mice tumours or OT cultures, after the combination treatment (Gemcitabine with ATRA). Scale bar 10 µm.

Supplementary Figure 12. ATRA alone or in combination with gemcitabine nuclear *Twist1* expression within cancer cells.

**A-D)** Representative images of tumour sections from *LSL-Kras^G12D/+^;LSL-Trp53^R172H/+^;Pdx-1-Cre* mice (KPC mice) stained with an anti-Twist1 antibody (red) and an anti-Cytokeratin antibody (green).

**E-H)** Representative images of Capan-1/PS1 organotypic (OT) sections stained with same antibodies as KPC mice sections. Scale bar 50 µm.

**a-d)** Zoom in images of the marked areas of KPC main images (A-D) with bold arrowheads pointing to nuclear *Twist1* expression in epithelial cells expression and empty arrowheads pointing to loss of this nuclear *Twist1* expression.

**e-h)** Zoom in images of the marked areas of OT main images (E-H) also with bold and empty arrowheads pointing to the differences of nuclear *Twist1* expression in epithelial cells upon different treatment conditions. ATRA alone or in combination with Gemcitabine reduces nuclear *Twist1* expression in epithelial cells, whilst in stellate cells nuclear *Twist1* expression remains unaltered. Scale bar 10 µm.

Supplementary Figure 13. The combination treatment affects the nuclear translocation of transcription factor *ZEB1* in cancer cells.

**A-D)** Representative images of tumour sections from *LSL-Kras^G12D/+^;LSL-Trp53^R172H/+^;Pdx-1-Cre* mice (KPC mice) stained with an anti-Zeb1 antibody (red) and an anti-αSMA antibody (green).

**E-H)** Representative images of Capan-1/PS1 organotypic (OT) sections stained with an anti-E-cadherin antibody (red) and an anti-Zeb1 antibody (green). Scale bar 50 µm.

**a-d)** Zoom in images of the marked areas of KPC main images (A-D) with bold arrowheads pointing to nuclear *Zeb1* expression within epithelial cells and empty arrowheads pointing to loss of nuclear *Zeb1* expression.

**e-h)** Zoom in images of the marked areas of OT main images (E-H) also with bold and empty arrowheads pointing to the differences of nuclear *Zeb1* expression in epithelial cells upon different treatment conditions. ATRA in combination with Gemcitabine reduces nuclear *Zeb1* expression in epithelial cells, whilst in stellate cells nuclear *Zeb1* expression remains unaltered. Scale bar 10 µm.

Supplementary Figure 14. The combination treatment affects the hedgehog signalling in cancer cells.

**A)** Representative images of tumour sections from *LSL-Kras^G12D/+^;LSL-Trp53^R172H/+^;Pdx-1-Cre* mice (KPC mice) stained by immunohistochemistry with an anti-Gli1 antibody. Zoom in images show the clear expression of nuclear and cytoplasmic Gli1 in ductal cells sections from untreated or Gemcitabine treated mice in comparison to a reduction of Gli1 expression in cancer cells of ATRA/Gemcitabine treated mice.

**B)** Representative images of OT sections stained by immunohistochemistry with an anti-Gli1 antibody. Zoom in images clearly show the reduction in Gli expression of cancer cells from Gemcitabine/ATRA treated OT cultures, which is in agreement with the differences in Gli1 expression also observed in KPC mice. Scale bar 100 µm. Zoom in images: Scale bar 10 µm.

**References**

**(Note: reference numbers correspond to reference list in main article)**

3. National Institute for Health and Care Excellence. The use of gemcitabine for the treatment of pancreatic cancer (TA25). 2001. Available from: https://www.nice.org.uk/guidance/ta25

11. Kadaba R, Birke H, Wang J, *et al*. Imbalance of desmoplastic stromal cell numbers drives aggressive cancer processes. *J Pathol* 2013; 230: 107–117.

14. Ene-Obong A, Clear AJ, Watt J, *et al*. Activated pancreatic stellate cells sequester CD8+ T cells to reduce their infiltration of the juxtatumoral compartment of pancreatic ductal adenocarcinoma. *Gastroenterology* 2013; **145:** 1121–1132.

30. Wong PP, Demircioglu F, Ghazaly E, *et al*. Dual-action combination therapy enhances angiogenesis while reducing tumor growth and spread. *Cancer Cell* 2015; **27:** 123–137.

41. Dangi-Garimella S, Krantz SB, Barron MR, *et al*. Three-dimensional collagen I promotes gemcitabine resistance in pancreatic cancer through MT1-MMP-mediated expression of HMGA2. *Cancer Res* 2011; **71:** 1019–1028.

**Supplementary Table 1. KPC mice characteristics at recruitment**

| **Treatment type** | **Age (days)** | **Tumour volume (mm^3^ on 2 days before treatment as measured by ultrasound)** |
| --- | --- | --- |
| **Control** | 249 | 137.761 |
| **Control** | 188 | 303.706 |
| **Control** | 170 | 326.367 |
| **Control** | 117 | 163.306 |
| **Control** | 120 | 195.04 |
| **Control** | 128 | 149.467 |
| **Gemcitabine** | 211 | 124.714 |
| **Gemcitabine** | 200 | 274.747 |
| **Gemcitabine** | 189 | 232.725 |
| **Gemcitabine** | 177 | 284.779 |
| **Gemcitabine** | 176 | 149.722 |
| **Gemcitabine** | 102 | 191.824 |
| **ATRA** | 216 | 156.138 |
| **ATRA** | 177 | 302.217 |
| **ATRA** | 243 | 327.712 |
| **ATRA** | 223 | 211.803 |
| **ATRA** | 150 | 160.042 |
| **ATRA + Gemcitabine** | 119 | 324.267 |
| **ATRA + Gemcitabine** | 171 | 234.933 |
| **ATRA + Gemcitabine** | 185 | 263.636 |
| **ATRA + Gemcitabine** | 218 | 495.181 |
| **ATRA + Gemcitabine** | 204 | 239.703 |
| **ATRA + Gemcitabine** | 124 | 343.271 |

**Supplementary Table 2. Table of antibodies**

| **Sections species origin** | **Antibody** | **Catalogue reference** | **Incubation period** | **Antigen retrieval method** | **IF (or IHC) dilution** |
| --- | --- | --- | --- | --- | --- |
| Organotypic sections  (anti-human) | Rabbit Cytokeratin | DAKO Z0662 | 1h, RT | HIER | 1:200 |
|  | Mouse Fibronectin | SIGMA F0916 | ON, 4^0^C | Pepsin | 1:100 |
|  | Mouse Ki67 | DAKO M7240 | 1h, RT | HIER | 1:100 |
|  | Mouse αSMA | DAKO M0851 | 1h, RT | HIER | 1:300 |
|  | Rabbit CC3 | Cell Signaling D175 | 1h, RT | HIER | 1:400 (IHC) |
|  | Rabbit Gli1* | Chemicon AB3444 | 1h, RT | HIER | 1:300 (IHC) |
|  | Mouse E-Cadherin | Abcam ab1416 | ON, 4^0^C | HIER | 1:100 |
|  | Rabbit RAR-β* | Abcam ab53161 | 1h, RT | HIER | 1:200 (IHC) |
|  | Mouse Twist1* | Abcam ab50887 | ON, 4^0^C | HIER | 1:100 |
|  | Rabbit Zeb1* | Santa cruz sc-25388 | ON, 4^0^C | HIER | 1:500 |
|  | Mouse FGF2* | Millipore 05-118 | ON, 4^0^C | HIER | 1:100 |
|  | Rabbit FGFR1* | Abcam ab10646 | ON, 4^0^C | HIER | 1:500 |
|  | Rabbit SFRP4* | Santa cruz sc-30152 | 1h, RT | N.A. | 1:50 (IHC) |
|  | Mouse Ezrin* | BD 10603 | ON, 4^0^C | HIER | 1:200 |
|  | Mouse β-Catenin* | BD 610154 | ON, 4^0^C | HIER | 1:200 |
| KPC mouse sections (anti-mouse) | Rabbit CK8 | Abcam ab59400 | ON, 4^0^C or 1h, RT | HIER | 1:100 |
|  | Rabbit Fibronectin | Abcam ab23750 | 1h, RT | HIER | 1:200 (IHC) |
|  | Rabbit Ki67 | Abcam ab15580 | 1h, RT | HIER | 1:150 |
|  | Mouse αSMA | SIGMA F3777 | ON, 4^0^C | HIER | 1:500 |
|  | Rabbit CC3 | Cell signaling  D175 | 1h, RT | HIER | 1:400 (IHC) |
|  | Rat Endomucin | Santa Cruz Sc-65495 | 1h, RT | HIER | 1:100 |
|  | Rabbit Glut1 | Millipore 07-1401 | ON, 4^0^C | HIER | 1:250 |

ON: overnight; 1h: one hour, RT: room temperature; HIER: Heat Induced Epitope Retrieval (citrate buffer pH 6); N.A.: not applicable.

*Used also in mouse.
